# An Artificial Therapist (Manage Your Life Online) to Support the Mental Health of Youth: Co-Design and Case Series

**DOI:** 10.2196/46849

**Published:** 2023-07-21

**Authors:** Aimee-Rose Wrightson-Hester, Georgia Anderson, Joel Dunstan, Peter M McEvoy, Christopher J Sutton, Bronwyn Myers, Sarah Egan, Sara Tai, Melanie Johnston-Hollitt, Wai Chen, Tom Gedeon, Warren Mansell

**Affiliations:** 1 Curtin enAble Institute Faculty of Health Sciences Curtin University Perth Australia; 2 Discipline of Psychology School of Population Health Curtin University Perth Australia; 3 School of Arts and Humanities Edith Cowan University Perth Australia; 4 Mental Health Commission of Western Australia Perth Australia; 5 Curtin Institute for Data Science Curtin University Perth Australia; 6 Centre for Clinical Interventions North Metropolitan Health Service Nedlands Australia; 7 Centre for Biostatistics School of Health Sciences The University of Manchester Manchester United Kingdom; 8 Alcohol, Tobacco and Other Drug Research Unit South African Medical Research Council Parow South Africa; 9 Division of Addiction Psychiatry Department of Psychiatry and Mental Health University of Cape Town Cape Town South Africa; 10 Department of Clinical Psychology School of Health Sciences The University of Manchester Manchester United Kingdom; 11 Mental Health Service Fiona Stanley Hospital Perth Australia; 12 Curtin Medical School Curtin University Perth Australia; 13 Centre of Excellence in Medical Biotechnology Faculty of Medical Science Naresuan University Phitsanulok Thailand; 14 Optus-Curtin Centre of Excellence in AI School of Electronic Engineering, Computing and Mathematical Sciences Curtin University Perth Australia

**Keywords:** mental health, conversational agents, chatbots, young people, acceptability, feasibility, co-design, artificial therapist, artificial intelligence, youth, child, adolescent, chatbot, Manage Your Life Online, MYLO, support, mobile phone

## Abstract

**Background:**

The prevalence of child and adolescent mental health issues is increasing faster than the number of services available, leading to a shortfall. Mental health chatbots are a highly scalable method to address this gap. Manage Your Life Online (MYLO) is an artificially intelligent chatbot that emulates the method of levels therapy. Method of levels is a therapy that uses curious questioning to support the sustained awareness and exploration of current problems.

**Objective:**

This study aimed to assess the feasibility and acceptability of a co-designed interface for MYLO in young people aged 16 to 24 years with mental health problems.

**Methods:**

An iterative co-design phase occurred over 4 months, in which feedback was elicited from a group of young people (n=7) with lived experiences of mental health issues. This resulted in the development of a progressive web application version of MYLO that could be used on mobile phones. We conducted a case series to assess the feasibility and acceptability of MYLO in 13 young people over 2 weeks. During this time, the participants tested MYLO and completed surveys including clinical outcomes and acceptability measures. We then conducted focus groups and interviews and used thematic analysis to obtain feedback on MYLO and identify recommendations for further improvements.

**Results:**

Most participants were positive about their experience of using MYLO and would recommend MYLO to others. The participants enjoyed the simplicity of the interface, found it easy to use, and rated it as acceptable using the System Usability Scale. Inspection of the use data found evidence that MYLO can learn and adapt its questioning in response to user input. We found a large effect size for the decrease in participants’ problem-related distress and a medium effect size for the increase in their self-reported tendency to resolve goal conflicts (the proposed mechanism of change) in the testing phase. Some patients also experienced a reliable change in their clinical outcome measures over the 2 weeks.

**Conclusions:**

We established the feasibility and acceptability of MYLO. The initial outcomes suggest that MYLO has the potential to support the mental health of young people and help them resolve their own problems. We aim to establish whether the use of MYLO leads to a meaningful reduction in participants’ symptoms of depression and anxiety and whether these are maintained over time by conducting a randomized controlled evaluation trial.

## Introduction

### Background

Globally, the prevalence of child and adolescent mental health issues has increased during the COVID-19 pandemic, up to a current rate of 1 in 5 [1]. Despite this increase, global government spending on mental health services remains low (2%), with shortages of skilled workers and a significant treatment gap between demand and provision for mental health disorders [2,3]. Digital interventions, including mental healthbased smartphone apps, that do not require guidance from mental health workers could be one solution for improving timely and equitable access to mental health support worldwide. Therefore, this paper reports the development of a mental healthbased smartphone app, Manage Your Life Online (MYLO), and assesses the acceptability and feasibility of this app to support the mental health of young people.

Several reviews have highlighted the benefits of using digital mental health apps (both on the web and offline) to improve consumer access to timely interventions by overcoming many traditional barriers to help seeking and enhancing therapeutic outcomes [4]. Mental health apps may be particularly well placed as a treatment option for adolescents and young people given the high levels of smartphone ownership worldwide [5-7] and initial reviews showing significant improvements in symptoms following app interventions [8]. Although apps provide an opportunity to reach youth who may have limited access to traditional mental health services, it is critical that such digital apps are theory driven, evidence supported, and highly engaging. However, a recent umbrella review (including 36 reviews conducted until 2022) found limited overall empirical and theoretical evidence for the efficacy of these apps or the therapeutic interventions they use [4]. Most apps use strategies based on therapy modalities and lack a theoretical underpinning or use >1 strategy or theory [9]. This makes it difficult to measure and draw conclusions on the most effective modality or theory to use in mental health apps and on how to improve mental health apps. Furthermore, limited user engagement and retention have been a pervasive issue across mental health apps [4,10], and this is largely driven by the user’s dissatisfaction with the functionality of the apps [11].

Conversational agents, or chatbots, that use artificial intelligence technology are a promising and fast-growing subset of mental health apps [12,13] that may be more engaging and therefore have higher levels of self-adherence than noninteractive apps [14]. Furthermore, as 71% of young people already report using messaging apps with peers to support their mental health, conversational agents can leverage users’ familiarity with texting to provide evidence-based support in a format with which users are already comfortable [15]. However, empirical evidence for the use of chatbots is currently lacking [14,16,17], and many apps are not designed and built according to a robust theoretical basis for a therapeutic paradigm [10,18]. For example, many use an eclectic mix of strategies (such as Tess [19], Wysa [20], and Shim [21]), and although this may offer users choice within the app, it becomes difficult to draw conclusions on which specific features and strategies are effective or not. Therefore, more research is needed to demonstrate the efficacy of conversational agents, including greater transparency and evaluation of the proposed mechanisms of action used [10,22].

Recent studies focusing on the user experience to identify ways to improve the uptake and engagement of mental health chatbots have generally found high user satisfaction [14,23]. Users have indicated that they value the interactive conversational approach and appear to build a relationship with the chatbots akin to that of a human therapist or friend [10,14]. These findings are consistent across chatbots that use a character or avatar for the agent (eg, Woebot [24], Wysa [20], and eSmart-MH [25]) and those that do not (eg, Tess [19]). Common challenges affecting conversational agents that may impact user engagement and satisfaction include repetitive content, limitations to the agent’s ability to understand the users’ expressed feelings or thoughts, inappropriate response to the user’s statements [10], and usability and technical issues [12].

Another challenge affecting engagement and efficacy of conversational agents, and mental health apps more broadly, is that many apps typically offer disorder-specific interventions rather than *transdiagnostic* (ie, effective for multiple mental disorders) or universal interventions. Universal interventions and apps use theories and therapeutic techniques that help reduce distress regardless of whether the symptom pattern or severity threshold conforms to those of a formal mental disorder (based on the narrow diagnostic criteria of the Diagnostic and Statistical Manual of Mental Disorders [26] or International Classification of Disease systems [27]) or the precise etiological factors driving the symptoms and impairments [28,29]. A universal approach could lead to increased user engagement and treatment efficacy by reducing the burden on users with multiple or overlapping comorbidities by removing the need to use multiple apps. Furthermore, universal interventions, both traditional and digital, have been shown to have similar effects on outcomes as their disorder-specific counterparts [30], yet are more flexible and scalable [31].

MYLO is an artificial intelligencebased conversational agent that emulates the method of levels (MOL) therapy [32], a universal therapeutic approach based on perceptual control theory (PCT), which is a unified model of psychological functioning [33-36]. According to PCT, psychological distress is caused by conflicting goals or values within an individual, and these internal conflicts lead the individual to experience loss of control, which manifests as psychological distress [33]. People have a hierarchy of different goals (values, ideals, and internal standards), with more important goals higher in the hierarchy and unresolved conflicts at higher levels entailing more chronic distress. According to PCT, an in-built learning process called reorganization can resolve conflict when a person’s awareness is sustained on the superordinate goal that drives the conflict. Therefore, therapeutic interventions based on PCT aim to sustain a client’s awareness of their problem to explore the conflict until a superordinate goal enters awareness, which is in turn explored to support the effective reorganization and restoration of control [32,37,38].

MOL therapists encourage clients to freely express and explore their problems by asking questions with appreciative curiosity to sustain a client’s attention to their problems and bring the client’s awareness to background thoughts that emerge while they are talking [32]. MYLO emulates MOL by asking users to describe their problem (eg, “I’m worrying about my daughter’s illness”), by identifying key terms and phrases in the users’ text (eg, “worrying”), and by selecting and generating an appropriate question based on these terms (eg, “What goes through your mind when you worry about this?”). By doing this, MYLO aims to provide a real-time personalized experience to users to help them explore their problems. Therefore, MYLO can address some of the challenges and recommendations previously mentioned regarding conversational agents.

An initial proof-of-concept randomized controlled trial (RCT) compared a single session of a MYLO prototype with a session with ELIZA, a chatbot that uses natural language processing to emulate a human-centered psychotherapist [39] with a university student sample [40]. MYLO was rated as more helpful than ELIZA, and participants in the MYLO group indicated significantly higher rates of problem resolution than those in the ELIZA group (*P*<.05). A similar, larger RCT with students and staff of 2 universities in the United Kingdom also found that MYLO was rated by users as more helpful than ELIZA [41]. Both studies found that participants reported reduced problem-related distress and reduced symptoms of depression, anxiety, and stress after using both chatbots. However, given that these studies used a single, approximately 20-minute session for university students and staff, clinically significant changes were not expected. A secondary aim of the study by Gaffney et al [40] was to test whether the mechanisms of change and reorganization of conflict described by PCT mediated participants’ helpfulness ratings and clinical outcomes. Indications of the mechanism were coded from the text conversations and were associated with greater distress reduction, improved problem resolution, and more positive expectations of using MYLO.

For the next stage of development, a MYLO prototype was provided for 2 weeks to a community sample of adults with self-reported diagnoses of anxiety or depression [31]. Participants identified the properties of MYLO that they found helpful, including providing a greater sense of control, a sense of being understood and respected, and being a good fit for the individual. The most helpful questions were those that allowed the user to talk freely and gain a new perspective or awareness of their problem.

Although participants have generally found MYLO to be an acceptable intervention, MYLO faces similar challenges to other chatbots, namely, ensuring that the content is appropriate and not repetitive [31]. To address these challenges and improve MYLO, participants from earlier studies made several suggestions for improving the MYLO interface, including modernizing it, using a more traditional messaging app layout, providing crisis contact information, and increasing the diversity and number of questions.

### This Study

In response to these recommendations, this study developed a new MYLO progressive web application (PWA) and interface. We recruited a youth advisory committee to help co-design this interface so that it would be accessible, engaging, and appropriate for young people aged 16 to 24 years experiencing symptoms of anxiety, depression, or low mood. To test the feasibility and acceptability of the new interface, we used a protocol similar to that of Gaffney et al [31] and gave participants the new MYLO app to test for 2 weeks, followed by qualitative interviews and focus groups. The results of this study will inform a second developmental stage that will include upgrading MYLO’s database and a fully powered RCT within this population. The specific aims are as follows:

1. Assess the feasibility of recruiting diverse young participants for a research study on MYLO
2. Assess MYLO’s acceptability and gain feedback on the research design
3. Assess the feasibility and acceptability of providing MYLO via a PWA to smartphone users aged 16 to 24 years
4. Assess the preliminary effects of MYLO on target outcomes for a future fully powered trial (eg, problem distress, anxiety, and depression symptoms) and the proposed mechanisms of change (eg, expressing oneself openly and freely and other tendencies toward the reorganization of goal conflict).

## Methods

### The MYLO Co-Design Phase

At the start of this research project, MYLO was available only as a web application. We recruited a youth advisory committee of 10 young people who had experienced anxiety or depression. A total of 7 committee members attended meetings or provided written feedback during the co-design phase. This group included 4 nonbinary people, 2 women, and 1 man, aged 16 to 24 years. Of these, 6 members lived in the Perth Metro area and 1 lived in a regional (ie, country) area of Western Australia. The panel was recruited by the lived experience researcher on the team through their existing networks and through the Consumer and Community Involvement program at the first author’s institute.

A total of 4 youth advisory committee meetings were held between July 1, 2022, and October 14, 2022, during which time the youth advisory committee tested different iterations of MYLO and provided feedback that was then presented to the software development team (Multimedia Appendix 1). The software development team implemented the committee’s feedback, and new iterations were then returned to the committee for further feedback.

### Ethics Approval

Approval for the case series was obtained from the Curtin University Human Research Ethics Committee (HREC2022-0466).

### Recruitment

A web-based digital advertisement was created and used to advertise the study between September 14, 2022, and October 21, 2022. The advertisement was shared by all members of the research group through their existing networks and personal social media pages. Twitter and Facebook profiles were also created for the MYLO app to advertise the study. The Twitter post shared by the MYLO Twitter profile was retweeted 22 times and gained 1731 impressions, and 15 clicks were gained on the survey link. A targeted Facebook advertising campaign was purchased for a 7-day period between October 12, 2022, and October 19, 2022, with a target audience limited to those in Western Australia aged 16 to 19 years, to recruit more participants aged 20 years. During this time, the advertisement reached 6275 people, resulting in 174 clicks on the survey link. During the recruitment period, several local and state-wide organizations, including consumer advocacy groups, mental health services, and other youth agencies, shared the advertisement either on social media or through their networks.

### Participants

Inclusion criteria were participants aged 16 to 24 years, currently living in Western Australia, having lived experience of anxiety or depression, having a smartphone and access to the internet, and being able to confidently read and type in English. Participants were also asked if they were able to commit to completing the web-based assessments each week (no more than 30 min/week) and were able to attend the 1-hour focus group after the testing phase. Participants were excluded if they were currently experiencing severe depressive symptoms or frequent suicidal thoughts. This was assessed using the Patient Health Questionnaire-9 [42], and participants who scored 20 (the established threshold for severe depressive symptoms) or scored 2 or 3 on the suicidal thoughts item (item 9) were excluded. All participants aged 18 years were asked if they wanted to provide their parents’ or guardians’ consent, and 3 of the 6 did.

We had several demographic targets to ensure that a wide range of young people were able to test and provide feedback on MYLO. These targets were a minimum of 2 men, 2 women, 2 people who identified as nonbinary, two 16- to 17-year-olds, two 18- to 21-year-olds, two 22- to 24-year-olds, 2 people who identified with a minority cultural group in Australia, and 2 people who lived in rural or remote regions of Western Australia (ie, not within the Perth or Peel metropolitan region). According to the Australian Bureau of Statistics [43], a minority cultural group in Australia is any group other than Australian, any of the North-West European groups, or any of the Southern European Groups (not including South Eastern and Eastern Europeans).

**Figure 1:**
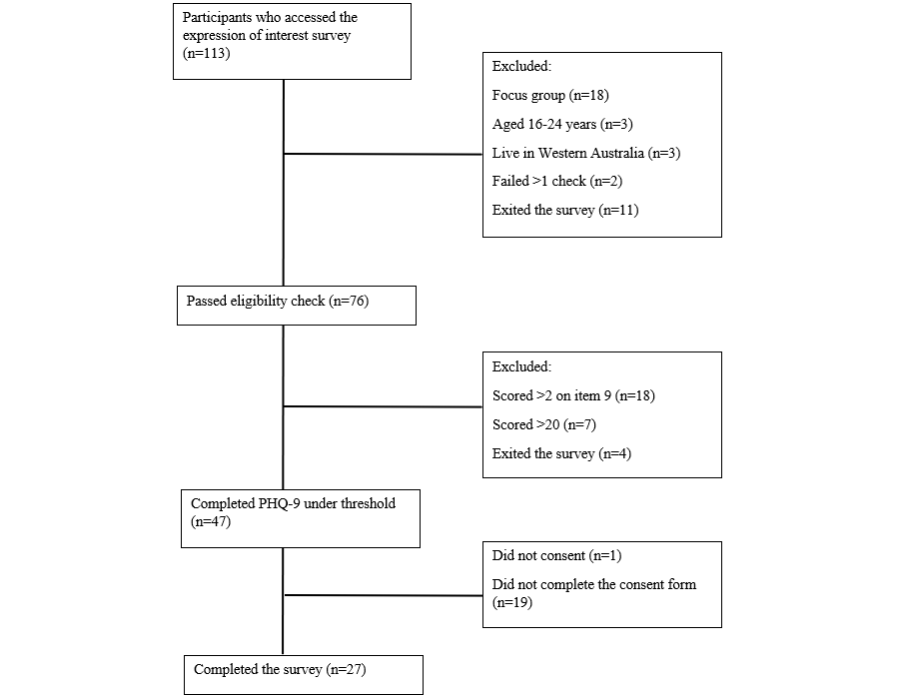
The number of participants excluded or lost through the expression of interest survey. PHQ-9: Patient Health Questionnaire-9.

Participants who followed the link or QR code on the advertisement were taken to an expression of interest survey hosted by Qualtrics (Qualtrics International Inc). The survey contained questions to ensure that participants met the inclusion and exclusion criteria, understood the study protocol, and provided informed consent and their contact details. Figure 1 shows the number of participants excluded or lost throughout this process. The research team reviewed the demographic information of the 27 eligible participants who completed the expression of interest survey and contacted a diverse range of young people. In total, 19 people were contacted to participate in the study; of these, 17 completed the baseline survey. A total of 4 participants were identified as completing the baseline survey from outside Australia, and their data were discarded, leaving a final sample of 13 participants.

### Materials

#### Web-Based Survey

Web-based assessments were administered via an anonymous survey hosted by Qualtrics at baseline, after 1 week of testing MYLO (during-testing survey), and after 2 weeks of testing MYLO (posttesting survey). Participants were sent an email or text containing the link to each survey as well as email or text reminders to complete the survey the following day. To link participants’ responses across the 3 time points while retaining anonymity, participants generated a subject-generated identification code [44]. Table 1 provides a summary of the self-report questionnaires included in the web-based assessments. Although we did not expect to see a significant change in these outcomes after 2 weeks of using MYLO, we calculated whether any participants experienced a reliable change in their scores over the 2 weeks. This was calculated using Cronbach α for each questionnaire and the reliable change method described by Evans et al [45]. To assess the acceptability of the questionnaire, participants were asked to rate how easy they thought each self-report questionnaire was on a 5-point scale, ranging from −2 (very difficult) to 2 (very easy), and participants could also provide qualitative feedback for each questionnaire via an open text box.

**Table 1:** The questionnaires used in the case series.

Questionnaire	Measures	Scoring
Patient Health Questionnaire-9 [42]	9 items; depression	0-4: minimal depression, 5-9: mild depression, 10-14: moderate depression, 15-19: moderately severe depression, and 20-27: severe depression.
Generalized Anxiety Disorder Assessment-7 [46]	7 items; anxiety	0-4: minimal anxiety, 5-9: mild anxiety, 10-14: moderate anxiety, and 15-21: severe anxiety.
General Health Questionnaire-12 [47]	12 items; psychiatric impairment	Traditional (acute) scoring method used. Scores range from 0 to 12, and higher scores indicate a greater possibility of psychological distress.
Short Form-6D version 2 [48]	6 items; general health	Scores range from −0.685 to 1, with 1 indicating perfect health. Australian weights were used for this sample.
Psychological Outcome Profiles [49]	4 items used for scoring; change in problem-related distress over the course of therapy	Scores range from 0 to 20. Decreases in score between pretherapy and posttherapy indicate that a positive change has occurred.
Reorganization of Conflict Scale [50]	10-item subscale; goal conflict awareness and the proposed mechanism of change in the method of levels therapy	Each item is scored from 0 (I do not believe this at all) to 100 (I believe this completely). The mean of the 10 items is used as the outcome.
General Self-Efficacy Scale [51]	10 items; self-efficacy	Scores range from 10 to 40. Higher scores indicate higher perceived general self-efficacy.
Session Impact Scale^a^ [52]	17 items; session (therapeutic) satisfaction	Each item is scored from 1 (not at all) to 5 (very much). We calculated the mean scores for the unwanted thoughts, relationship impacts, hindering impacts, understanding, and problem-solving subscales. Item 17 measures “other impacts,” an optional item that is not used in scoring.
System Usability Scale^a^ [53]	10 items; user experience of digital systems	Outcome is a percentile ranking from 0 to 100, with scores >68 considered above average.
User Engagement Survey^a^	3 items; how often and for how long participants used Manage Your Life Online	Users select the days they used MLYO (Monday to Sunday), estimated how long an average conversation lasted (in mins), and how many conversations they had on each day they used MYLO.

^a^Denotes surveys that were only presented at the during-testing survey and post-testing survey.

#### Manage Your Life Online

**Figure 2:**
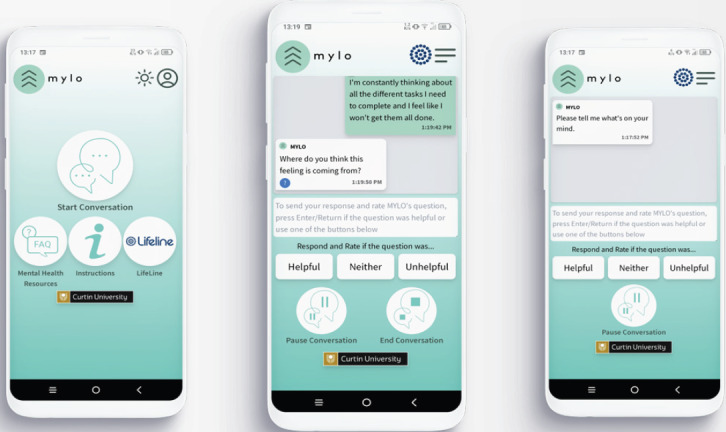
Screenshots of the Manage Your Life Online (MYLO) progressive mobile app interface.

MYLO was provided as a PWA that could be accessed through a web browser and downloaded onto the user’s smartphone (Figure 2). From the home page, users could choose to start a new conversation, resume their last conversation, or access a range of mental health resources. When a new conversation commences, MYLO asks the user “Please tell me what’s on your mind.”, users are then able to type free text about the problem they would like to explore. MYLO analyzes users’ text for key terms (eg, “anxious”) and phrases (eg, “can’t sleep”) and responds with a question (eg, “What do you think about feeling anxious?”). These questions are designed to emulate the questions that an MOL therapist would use [32] and aim to prompt users to consider their problems from a higher level of awareness. By doing so, users can become better at resolving their problems and, therefore, reduce the level of problem-related distress they experience [32]. The conversation continues with MYLO asking questions and the user responding until the user chooses to end the conversation.

Within the interface, users also have access to a list of mental health resources as well as a button that connects them to the Lifeline call center—an Australian suicide prevention hotline. These resources were included to provide users with the ability to connect to face-to-face or crisis services if they feel they need to. Users also have limited ways to customize their profile by changing their profile name and the colors of their avatar (their initial on a colored square). Both features were recommended by the youth advisory panel to improve safety and acceptability, respectively. MYLO uses built-in control systems to identify relevant terms in users’ responses and to generate an appropriate question in response, and it uses these systems to improve at both tasks. Users rate each of MYLO’s responses, which generates an error term for each unique term and question pairing as well as each question and term on its own. Each question, term, and question and term pairing started with an error term of 0, meaning they are “helpful” at the beginning of the testing phase. The more a question and term are rated as unhelpful or neither helpful nor unhelpful, the larger their error terms become (with higher error values being added for unhelpful ratings compared with neither helpful nor unhelpful ratings). Equally, the more questions and terms are rated as helpful, their error terms are reduced. Once a question and term pairing has been used >5 times, MYLO uses the error terms to sort its list of possible questions when selecting the best questions, making it less likely that unhelpful questions will be selected and more likely that helpful questions will be selected. It was decided the pairing needed to be used 5 times before learning begins to ensure that error terms were based on a pattern of helpfulness, as a question may be helpful to one person but unhelpful to others. This information was used to examine the engagement of participants with MYLO, explore the acceptability of MYLO’s questioning, and judge whether MYLO can learn and thereby adjust its questioning in the future based on the ratings given by the participants.

#### Focus Group

The topic guide (Multimedia Appendix 2 [31,54]) was adapted from the study by Gaffney et al [31] to investigate what the participants found helpful and unhelpful regarding MYLO. Other questions were adapted from the study by Ly et al [54] to gauge the engagement of participants and use of MYLO. Participants were also asked about their experience of completing the web-based assessments to examine the acceptability of the measures used for future studies. The focus group was recorded and transcribed using an independent local transcription service. Inductive content analysis of the transcripts was conducted by the first author according to the steps described by Vears and Gillam [55]. The coding schemas were discussed with the last author and refined.

### Procedure

All participants were provided with the newly developed MYLO PWA to test for 2 weeks. During this time, participants completed 3 web-based assessments: at baseline, after 1 week of testing MYLO (during-testing survey), and after 2 weeks of testing MYLO (posttesting survey). The assessments contained several self-report questionnaires on psychological well-being and experience with the MYLO app. After the 2-week testing phase, participants attended a web-based focus group to provide qualitative feedback on their experience with the MYLO PWA and the study protocol. Participants received digital gift vouchers of Aus $20 (US $13.40) per hour (maximum of 4 hours) for their time testing MYLO, completing the web-based assessments, and attending the focus group.

## Results

### Recruitment and Retention

The final sample consisted of 13 participants who completed the baseline survey. The final sample met all the diversity targets for gender, age, cultural group, and region (refer to the *Methods* section for more details). The demographics of the participants are summarized in Table 2. Of the 13 participants, 10 (77%) completed all web-based surveys, and the 10 participants provided qualitative feedback (n=5, 38% participants attended a web-based focus group and owing to limited availability, n=3, 23% attended web-based interviews, n=1, 8% provided written feedback to the focus group questions, and n=1, 8% provided brief feedback via email). A total of 15% (2/13) of participants dropped out in the first week of testing (ie, they did not complete the during-testing survey), and neither of these participants gave a reason. The participants who did not complete the final survey informed the researchers that they were too busy; this was also the same participant who provided brief feedback via email. Another participant who provided written feedback rather than attending an interview informed the researchers that they were unwell while the focus groups and interviews were being conducted and therefore could not attend the focus groups and interviews.

**Table 2:** Participant demographics.

ID	Gender	Age group (years)	Cultural group	Region
1^a^	Nobinary^b^	16-17	Australian, *Malay*^b,c^	Metro^d^
2^a^	Nonbinary^b^	16-17	Australian, English	Metro
3	Nonbinary^b^	18-21	Australian	*Regional*
4	Woman	16-17	Australian, *Chinese*	*Regional*
5	Woman	16-17	Australian	*Regional*
6^a^	Woman	16-17	Italian	Metro
7^a^	Woman	16-17	Australian, Scottish	Metro
8^a^	Woman	18-21	Australian, English	Metro
9^a^	Woman	22-24	*Filipino*	Metro
10	Woman	22-24	Australian	Metro
11^a^	Man	18-21	Australian	*Regional*
12	Man	22-24	Australian	*Regional*
13^a^	Man	22-24	Australian, Scottish	Metro

^a^Denotes participants who attended a focus group or interview.

^b^Denotes where a participant used a self-describe textbox.

^c^Italicization indicates cultural groups and regions that met the diversity targets.

^d^Metro: metropolitan.

We retrospectively collected information on the sample’s sexuality to further assess the diversity of the sample, and of those who disclosed their sexuality, 3 described themselves as heterosexual, 1 as lesbian, 1 as pansexual, and 1 as “vincian/gay (attracted to men and masc. [gender] people).” Participants completed an anonymous survey during the second week of testing MYLO and were asked to self-describe their sexuality. In the future, this information will be gathered during the expression of interest survey.

### Acceptability of the Research Design

#### Web-Based Survey

The difficulty ratings for all the surveys are provided in Multimedia Appendix 3. None of the questionnaires received a negative mean score. The lowest ratings were for the Session Impact Scale (SIS), measuring therapeutic satisfaction (mean 0.3, SD 1.06), and the engagement questionnaire (mean 0.4, SD 1.17), indicating that both were of “neutral” difficulty to complete. The engagement questionnaire was also the only questionnaire to be rated very difficult by 1 participant who explained in the textbox that they had severe memory problems because of a psychological disorder and, therefore, could not remember when they used MYLO during the week. All other questionnaires received mean scores of 0.6 to 0.8, indicating that participants found them neutral to complete. Some participants who completed the web-based difficulty ratings (10/13, 77%) also provided qualitative feedback in the survey (4/10, 40%), with 1 stating that they preferred the Patient Health Questionnaire-9 (depression) style questions to the Short Form-6D version 2 (SF-6Dv2) General Health Questionnaire style questions, although they rated both as very easy. Another participant suggested it would be useful to ask about life events that are impacting the participants to better understand why their scores may have changed during the testing period:

Possibly a useful thing is asking about the context? i.e., Has anything changed in the past few weeks that we should take into consideration when we are evaluating this survey?

Finally, 1 participant used the textbox to state that their health had deteriorated during the testing phase but that it was not MYLO’s fault:

MYLO not helping was not MYLO fault. Bad health and stuff get worse. MYLO did not make it worse.

Participants were also asked to rate the overall survey length. Of the 10 participants who completed the posttesting survey, 7 said the survey was too long and 3 said it was about right (no one said it was too short).

#### Qualitative Feedback on the Intervention

In focus groups or interviews, participants were positive about their experience of participating in the MYLO study, and some indicated they would be interested in participating again. Length of the surveys and testing time frames were both found to be acceptable. Most participants (7/8, 88%) found the surveys easy to complete, and none of the questions or surveys were flagged as distressing, although some participants (3/8, 38%) described the surveys as “samey” or repetitive. Despite the time commitment, several participants indicated that they saw the value of participation and were happy to contribute. Recommendations and technical issues were also reported to the team and are detailed in Textbox 1.

**Participant recommendations to improve the research design.**

- Change the testing time frame so that surveys are completed every 2 weeks.
- Surveys should take a maximum of 15 minutes.
- Conduct short qualitative interviews midway through the testing phase.
- Monitor life events during the testing phase.
- Should be able to pause and resume completing the survey over several sittings.
- One scale (reorganization of conflict) required the participants’ phone to be in landscape mode.
- The slider on the Reorganization of Conflict Scale (0-100) should be changed to a Likert-type scale like the other surveys.

### Feasibility and Acceptability of MYLO

We assessed the feasibility and acceptability of MYLO across 3 categories: engagement with MYLO, acceptability of the interface, and acceptability of MYLO’s therapeutic conversations.

#### Engagement With MYLO

Participants reported using MYLO between 1 and 4 days a week in the first week and having 1 to 3 conversations with MYLO on those days. Participants reported using MYLO for a variety of reasons: when they needed to share or talk about something, when they felt low, and when they had spare time. Several participants attributed their drop in use in the second week to MYLO’s repetitive questioning. Another participant said they forgot about MYLO, and this contributed to their lower use:

Because it didn’t become, like, part of my routine that I do all the time, it just...I’d forget that it was a thing.

Nonbinary, 16-17 years

The average length of a conversation ranged from 2 to 30 minutes, with most conversations lasting 10 to 15 minutes (n=8). Of the participants who provided conversation length for each day (n=9), the total time of using MYLO over the week ranged from 7 to 62 minutes, with most participants using MYLO for 30 to 35 minutes (n=6). In the second week, participants reported using MYLO between 1 and 7 days and having 1 to 5 conversations with MYLO on those days. The average length of a conversation ranged from 5 to 15 minutes. Of the participants who provided conversation length for each day (n=7), the total time spent using MYLO over the week ranged from 15 to 40 minutes.

#### MYLO Use Data

Only conversations with 1 response were included in this analysis. The participants had 32 conversations with MYLO between October 17, 2022, and November 4, 2022. This time is longer than 2 weeks as recruitment of participants was staggered; the final participant finished the 2-week testing phase on November 7, 2022. The character count of these conversations ranged from 58 to 2104 characters including spaces and participants sent 2 to 20 texts. A total of 13 conversations had 5 participant texts, 11 had between 6 and 10 participant texts, and 8 had between 11 and 20 participant texts. Participants used MYLO at various times of the day: 8 of them used it between midnight and 6 AM, 12 between 6 AM and noon, 12 between noon and 6 PM, and no one used MYLO between 6 PM and midnight. The texts sent by participants included 23 different themes (this does not include the themes from the 13 conversations that were 5 responses, as MYLO does not currently record this information; this also only includes themes that were used to choose a question; other themes may also have been present in texts sent by participants) drawn from 48 unique terms (refer to Multimedia Appendix 4 for the full list).

**Figure 3:**
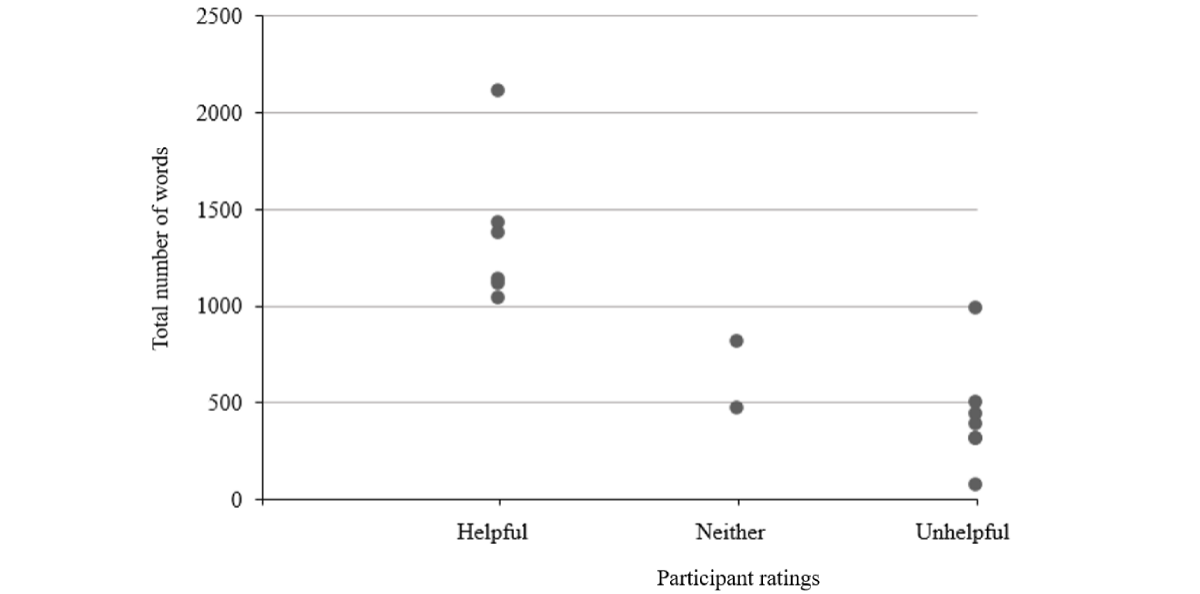
Number of participant-generated characters in each conversation and overall helpfulness rating.

Participants rated 15 conversations: 6 were rated as helpful, 2 as neither helpful nor unhelpful, and 7 as unhelpful. As shown in Figure 3, in total 100% of the conversations in which participants typed over 1000 characters were rated as helpful, and the remaining conversations were rated as either unhelpful or neither.

At the end of the testing phase, 61 unique questions and term pairings were rated 75 times by the participants, including 40 unique questions. Table 3 provides a summary of the questions used more than once and the ratings they received during the testing phase. Of the 61 question and term pairings, 41 (67%) had an error term of 0 at the end of the testing phase, indicating that the pairings (and the questions and terms in the pairings) were only ever rated as helpful. The remaining pairings had various error terms 0, indicating that they received ratings other than helpful. The differences in error terms indicate that MYLO records user ratings of the questions and uses this feedback to adjust its learning system.

**Table 3:** Questions used more than once by Manage Your Life Online and their helpfulness ratings (n=75)^a^.

Questions	Total, n (%)^b^	Helpful, n (%)^c^	Neither, n (%)^c^	Unhelpful, n (%)^c^
“When you feel^“d”^, what goes on in your body or in your mind?”	9 (12)	6 (67)	1 (11)	2 (2)
“What makes you use the word^“d”^?”	7 (9)	5 (71)	1 (14)	1 (14)
“How do you think you could begin to do that?”	4 (5)	4 (100)	0 (0)	0 (0)
“Where do you think this feeling is coming from?”	4 (5)	4 (100)	0 (0)	0 (0)
“When you say^“d”^, how does that actually feel for you?”	4 (5)	3 (75)	0 (0)	1 (25)
“You are saying that you want to do something. What is getting in the way?”	3 (4)	2 (67)	0 (0)	1 (33)
“When you say^“d”^, how often do you feel like this?”	3 (4)	3 (100)	0 (0)	0 (0)
“How is this feeling affecting you?”	3 (4)	3 (100)	0 (0)	0 (0)
“Tell me more about what you would like?”	3 (4)	2 (67)	1 (33)	0 (0)
“How do you feel about looking at the future like that?”	3 (4)	2 (67)	1 (33)	0 (0)
“What would help you achieve that?”	2 (3)	2 (100)	0 (0)	0 (0)
“What thoughts about yourself are associated with^“d”^?”	2 (3)	2 (100)	0 (0)	0 (0)

^a^Total number of questions rated by participants during the testing phase.

^b^Percentage of the total number of questions rated by participants during the testing phase.

^c^Percentage of times the question was rated by participants during the testing phase.

^d^Text in quotation indicates the term that was identified in the users’ text.

#### Acceptability of the Interface

Participants rated the usability of the MYLO interface during testing and posttesting using the System Usability Scale. The mean rating across both time points was 73.57 (SD 16.02) and ranged from 37.50 to 97.50 (median 77.5). Scores increased slightly between the during-testing survey (mean 71.59, SD 16.17) to the posttesting survey (mean 75.75, SD 16.42). The overall mean indicates that MYLO is better than the average of other systems (median score 68 [56]) and is currently ranked within the 65th to 69th percentile of user systems, giving it a grade of B—meaning it is “acceptable” [56]. Single-question scores were examined to determine which areas could be targeted for improvement. Participants only scored 2 questions below average—questions 1 and 5: “I think that I would like to use this system frequently” and “I found the various functions in this system were well integrated,” respectively.

Participants were able to provide brief qualitative feedback after completing the System Usability Scale as well as during the focus groups and interviews regarding the MYLO interface. The participants said that the interface’s simple design made it easy to use. Participants also liked the colors and that MYLO was being developed locally. The participants made recommendations for MYLO, which are reported under MYLO recommendations in the *Results* section. Several technical issues were reported but none seemed to cause participants to disengage from using MYLO.

### Acceptability of MYLO’s Therapeutic Conversations

#### Overview

The acceptability of MYLO’s therapeutic conversations with the user was assessed using the participants’ therapist satisfaction scores and focus group and interview data. First, we report participants’ satisfaction with MYLO’s text-based conversations, followed by their satisfaction with the conversations as a therapy session and which aspects of the conversations they felt were useful. Finally, we report the difficulties reported by the participants and their recommendations to improve MYLO.

#### Satisfaction With MYLO’s Text-Based Conversations

Most participants expressed satisfaction with their conversations with MYLO and liked the text-based conversation system, explaining that it allowed them to access support discretely without being judged and in different situations. Some participants (2/8, 25%) expressed preferring texting to talking about their feelings:

I prefer, like, texting and getting my feelings out. Just because I can really quickly, like, my fingers catch up to my brain. So, I just prefer the typing.

Woman, 18-21 years

One interview participant had issues with verbal expression and memory that had previously negatively impacted in-person therapy:

I have some speech issues. So, like, being able to type is a lot easier for me. And it was really good to be able to, like, because that’s an issue that I’ve had with regular therapy as well, like, being able to verbally express. So being able to type everything out was really helpful. So, it was really good in terms of the typing,

Verbal expression issues, nonbinary, 16-17 years

It would be easy to, like, read back, like, see what I’d said, see what MYLO said, because sometimes, like, in the middle of conversations, I just forget everything, so I have to, like, refresh myself, where was I? And so, it’s really good for that. Like, if I’m in the middle of something, and, you know, we need to go back and get more context, I can. So that’s really helpful, because, again, it was quite a barrier when I was doing in-person therapy where, like, I’d suddenly forget everything in the middle of the session, and I’d have to be, like, “Can you tell me again what we were talking about?”

Memory issues, nonbinary, 16-17 years

#### Satisfaction With Therapy Sessions

The mean therapy satisfaction scores across participants were compared with the existing cohorts of participants (Table 4) with anxiety and depression receiving computerized therapy [57] and brief in-person psychological interventions [58,59]. It is worth noting that the participants completed these measures during the weekly surveys rather than after every session with MYLO.

**Table 4:** Session impact subscale scores for Manage Your Life Online (MYLO) and other psychological therapiesa.

Session impact subscale	MYLO	Computerized cognitive behavioral therapy [60]	Therapist-delivered cognitive behavioral therapy [60]	Psychotherapy [58]	Psychodynamic therapy [57]	Cognitive behavioral therapy [57]
Understanding, mean (SD; 95% CI)	2.43 (1.00; 2.00-2.86)	2.35 (0.49; 1.92-2.78)	3.03 (0.82; 2.23-3.83)	2.60 (1.05; 2.55-2.65)	2.87 (0.71; 2.64-3.10)	2.73 (0.77; 2.48-2.98)
Problem-solving, mean (SD; 95% CI)	2.14 (1.05; 1.69-2.59)	2.79 (0.76; 2.12-3.46)	3.44 (1.00; 2.46-4.42)	2.87 (1.11; 2.82-2.92)	2.79 (0.64; 2.59-2.99)	3.36 (0.67; 3.14-3.58)
Relationship, mean (SD; 95% CI)	2.28 (0.93; 1.88-2.68)	2.62 (0.64; 2.06-3.18)	3.43 (0.89; 2.56-4.30)	3.11 (1.04; 3.06-3.16)	3.22 (0.74; 2.99-3.46)	3.28 (0.75; 3.04-3.53)
Hindering, mean (SD; 95% CI)	1.77 (0.58; 1.52-2.02)	1.19 (0.10; 1.10-1.28)	1.14 (0.15; 0.99-1.23)	1.17 (0.37; 1.15-1.19)	1.20 (0.26; 1.12-1.28)	1.14 (0.28; 1.05-1.23)
Unwanted thoughts, mean (SD; 95% CI)	1.52 (0.75; 1.20-1.84)	1.35 (0.30; 1.09-1.61)	1.46 (0.32; 1.15-1.77)	1.50 (0.83; 1.46-1.54)	1.51 (0.44; 1.37-1.65)	1.47 (0.49; 1.31-1.63)

^a^Session impact subscale score: 1=not at all, 2=slightly, 3=somewhat, 4=very much, and 5=very much.

Comparison with previous studies suggests that MYLO scored slightly lower on understanding (except when compared with computerized cognitive behavioral therapy [57]), problem-solving, and relationship than the other interventions and slightly higher on hindering impacts. Participants experienced similar unwanted thoughts after using MYLO as after using other interventions.

The individual scores across the 5 subscales varied (Multimedia Appendix 5). For example, individual means for understanding and problem-solving (2 subscales that most closely align with MYLO’s proposed mechanisms of change) ranged from 1 to 4. For understanding, 7 participants had a mean of 2, indicating that their sessions with MYLO were at least slightly helpful in supporting them to gain understanding. Similarly, during the focus groups and interviews, many participants described MYLO as helpful and suggested that they were able to gain some insight into themselves or their problems while using it:

Yeah, no, it taught me, like, quite a bit about myself in, like, the short timeframe, so it is a really useful tool.

Woman, 16-17 years

The participants said that MYLO made them consider and explore their problems by asking novel questions. Even participants who acknowledged that this was usually a difficult task for them described the process of exploration with MYLO as helpful:

I think it really helped me capture or, like, kind of explore how I felt because usually, what happens is for me, when a problem comes, all my emotions are wrapped in a bundle and it’s hard for me to unravel that, or express or process that. So I think that was helpful.

Woman, 22-24 years

Some participants (2/8, 25%) stated that since using MYLO they have continued to think about their problems following MYLO’s principles, even when the conversation with MYLO might have ended poorly:

But I’ve noticed, even when I’m not using MYLO, it kind of helps ground me when I’m, like, oh, I need to think about why I’m feeling this way. So, all in all, it has helped me, even today.

Woman, 16-17 years

I found even when I left the conversation, feeling, like, kind of annoyed, I noticed that I would still keep picking up things that MYLO has, like, taught me, especially with kind of trying to understand why I’m feeling something or exactly what I’m feeling, and kind of bringing myself back down to the ground.

Woman, 16-17 years

Although the comparison of SIS scores suggested that MYLO performed slightly worse than in-person therapy, for some participants (3/8, 38%), the lack of a human therapist improved their experience, as they did not feel judged:

Yeah, and especially because it’s an app, like, I don’t feel judged by anyway. Like, I know, it’s anonymous.

Woman, 18-21 years

A participant felt that the process was less overwhelming:

Yeah, I think sometimes the presence of someone, like, across from you is, like, overstimulating sometimes, so everything’s, like, going on at once.

Woman, 16-17 years

#### Difficulties With MYLO Conversations

The SIS scores suggest that all participants experienced some difficulties with MYLO’s conversations, impacting its helpfulness and, in some cases, causing frustration. Some participants (3/8, 38%) felt that MYLO had difficulties understanding them because of how they were typing (ie, number of words and content of the message). Participants who experienced this problem adjusted the language they used, and the problem was resolved:

I had a little bit of an issue at first where I asked, like, I said something specific and it didn’t understand, but once I was using it more, I understood, like, to use broader words, stuff like that.

Woman, 18-21 years

Some participants (4/8, 50%) also found it difficult to explain their feelings:

I found it difficult when it would ask to kind of, like, explain, like, in a few more sentences what you were feeling because I’m not much of a talker.

Woman, 16-17 years

Another barrier for participants initially engaging with MYLO and having a successful conversation was their internal state. Participants who were distressed did not want to or did not have the capacity to explore their problems.

The largest problem that caused participants to disengage with MYLO during a conversation was the repetition of questions or the use of very similar questions that made participants feel they were repeating themselves:

That’s why I walked away frustrated, just because it said the same things, and then I didn’t want to have to re-explain myself. Like, I don’t want to expand on what I said because I’ve already just said it.

Woman, 18-21 years

Questioning was also described by 1 participant as overwhelming. Finally, some participants (4/8, 50%) also had trouble understanding some of MYLO’s questions, so they struggled to answer them:

I don’t know, I sort of struggled with, like, the questions that MYLO asked though, like, I sort of struggled to understand most of them, like, what they were sort of wanting me to talk about, I guess.

Nonbinary, 16-17 years

When asked to elaborate, the participant described the questions as vague and gave an example:

It would ask me, like, why I said the word “stressed” about something...which I didn’t really know how to answer.

Nonbinary, 16-17 years

These questions are typical of MOL therapy, where a therapist will inquire about the language or words people use to encourage them to explore their experience without the therapist assuming they understand the client’s experience based on the language a client used [61]. This may be challenging for some users, especially if they have not attended an MOL session before. These types of questions also seem to be those rated “unhelpful” most often (Table 3) and therefore will need to be improved in future development stages.

#### MYLO Recommendations

Participants provided recommendations to address some of these issues and improve other aspects of user experience (Table 5). The suggestions included changes to the MYLO interface that provided more control to the user over the aesthetics of the interface, such as options to customize the colors, changes to the MYLO database (eg, a larger range of questions), and additional features (eg, mindfulness or grounding techniques) to help participants get into the right headspace to use MYLO by reducing their initial distress.

To fulfill the recommendation to save old conversations, participants need to be able to create a unique user profile and log in. Case-series participants were, therefore, asked about different methods of achieving this and their preferences. Participants did not reach a consensus on how best to achieve this, but it was important to all of them that logging in and accessing MYLO remained easy and straightforward. Many participants expressed concerns about remembering passwords or other log-in credentials, especially if they were in an emotional state when they wanted to talk to MYLO. Some participants (2/8, 25%) were concerned about data privacy and indicated that they would want to be advised on how and what data were being stored.

**Table 5:** Participants’ recommendations to improve Manage Your Life Online (MYLO).

Recommendations	Participants (n=10), n (%)
Participants sometimes forgot to talk to MYLO; hence, they would like notifications to use MYLO that they could control the frequency of.	6 (60)
Participants wanted to be able to revisit the previous conversations and would like to save old conversations, or sections of conversations.	7 (70)
Participants wanted more control over the look of the app and a way to make it feel like their own space, such as customizable color schemes.	4 (40)
MYLO has a “Resume/Pause conversation” button, but some participants experienced issues with this system and would like it to be improved.	4 (40)
Participants wanted a native app that was easier to download and access through their smartphones.	3 (30)
Participants wanted the option of using speech-to-text to improve their ability to express their feelings or problems.	3 (30)
Participants wanted the ability to text multiple times in a row rather than having MYLO respond after each message to suit their natural texting behaviors more closely.	3 (30)
Participants wanted the app to include mindfulness and grounding techniques that they could use if they were too distressed to talk with MYLO.	3 (30)
Participants wanted some strategies to be recommended for the recurrent problems they discussed with MYLO.	2 (20)
Participants suggested having rotating backgrounds similar to Windows to improve the aesthetics of MYLO.	2 (20)
Participants wanted an instructional demonstration of how to use and talk with MYLO to improve its usability.	2 (20)
One participant wanted the ability to use MYLO offline, improving MYLOs usability and accessibility.	1 (10)
One participant suggested MYLO be able to use and recognize emojis to communicate with young people more naturally.	1 (10)
One participant suggested a space in the app to record or vent without MYLO asking questions.	1 (10)
One participant suggested a cross platform profile so they could use MYLO on any device and access their previous or paused conversations.	1 (10)
One participant suggested the ability for MYLO to connect users with a person or expert in the app to receive human support.	1 (10)
One participant suggested a larger repertoire of questions to reduce repetition.	1 (10)
One participant requested access to peer support within the app.	1 (10)
One participant wanted examples of how to answer questions in the conversation window.	1 (10)
One participant suggested MYLO be able to check on users’ well-being during the conversation to ensure they are safe to continue.	1 (10)
One participant said the “Helpful/neither/unhelpful” buttons needed to be clearer, both what their purpose is and their visibility on the screen.	1 (10)
One participant suggested that MYLO should be able to give positive feedback during conversations when a user is doing well or progressing.	1 (10)

Participants in each focus group and interview were also asked whether they would recommend MYLO. Of the 8 participants, 6 (75%) would recommend MYLO and the remaining 2 (25%) would recommend MYLO with some improvements. The reasons for participants to recommend MYLO were as follows: MYLO is easy to use (n=2), MYLO is easy to access (n=2), traditional psychological support is expensive (so MYLO would ideally be free, n=2), and MYLO is a good supplementary tool (n=1). One participant said that they knew friends who liked to work through their problems in a similar way:

I'll definitely be recommending it to my friends and stuff. Because a lot of them process issues the same way I do where you sort of need to, like, talk it out and figure things out for yourself. So, it'd be really helpful for them as well.

Nonbinary, 16-17 years

One participant thought it would be particularly useful for young people, and another participant said it would be useful for those who do not feel comfortable accessing in-person therapy and are experiencing milder symptoms.

### Target Outcomes

As the current sample was small and the testing time frame was short, we did not expect to observe significant improvements in the participants’ clinical outcomes. Table 6 presents the mean scores over time. Cohen *d* was calculated for each outcome at 2 weeks relative to baseline and showed at least a small effect (ie, Cohen *d*≥0.2) for each domain, except for general health, depression, and self-efficacy. The sample’s problem-related distress scores were further examined by calculating the Psychological Outcome Profiles effect size, which provides an estimate of the effect size of change for the sample between baseline and posttesting (ie, pretherapy and posttherapy Psychological Outcome Profiles scores; this is calculated by subtracting the mean posttherapy score (posttesting) from the mean pretherapy score (baseline) and dividing the result by the SD of the pretherapy score). In this sample, the effect size was 1.50, indicating a large effect size [59].

Table 7 shows individual changes in scores from the baseline survey to the posttesting survey for participants who completed the measures at both time points. The reliable change index was calculated for each participant on each outcome, and those that were found to have reliably changed are denoted in Table 7. A total of 3 participants reliably deteriorated on a single measure during the testing phase: 1 participant’s general health (SF-6Dv2), 1 participant’s anxiety (Generalized Anxiety Disorder Assessment-7), and 1 participant’s self-efficacy (General Self-Efficacy Scale). Inspection of the participant’s SF-6Dv2 results showed a 1-point deterioration in their scores for physical functioning, body pain, vitality, and mental health between the baseline and posttesting surveys. A total of 7 participants experienced reliable improvements during the testing survey, and at least 1 participant improved in each outcome, with 1 participant improving across all outcomes.

**Table 6:** Mean scores on clinical outcomes at baseline, during, and after testing Manage Your Life Online for 2 weeks.

Outcome	Baseline survey (n=13), mean (SD)	During-testing survey (n=11), mean (SD)	Posttesting survey (n=10), mean (SD)	Change^a^, mean (SD; 95% CI)	Cohen *d*
General health	0.51 (0.24)	0.32 (0.85)	0.43 (0.39)	−0.02 (0.29; −0.19 to 0.15)	0.07
Depression	11.39 (3.82)	10.27 (3.90)	10.80 (4.96)	−0.10 (3.73; −2.29 to 2.09)	0.03
Anxiety	9.54 (4.18)	7.73 (4.52)	8.00 (4.06)	−1.40 (4.65; −4.13 to 1.33)	0.39
Psychiatric impairment	6.00 (3.46)	5.09 (3.15)	6.20 (4.24)	−0.80 (3.71; −2.98 to 1.38)	−0.23
Goal conflict reorganization	63.42 (16.07)	66.62 (14.42)	72.54 (13.73)	8.88 (11.59; 2.07 to 15.69)	−0.66
Self-efficacy	26.54 (3.87)	26.55 (4.39)	26.90 (3.57)	0.50 (3.57; −1.60 to 2.60)	−0.16
Problem-related distress	14.23 (2.28)	12.82 (2.64)	10.90 (3.60)	−3.70 (4.19; −6.16 to −1.24)	1.26

^a^The change column presents mean change between baseline and posttesting survey scores; therefore, the scores of the participants who did not complete the posttest survey were not included.

**Table 7:** Change from the baseline survey score to the posttesting survey score.

Outcome	Cronbach α	Reliable change index [45]	Participant^a^									
			1	2	3	4	5	6	7	9	10	11
General health	.76	0.32	−0.09	0.24	−0.15	0.28	−0.29	0.27	0.34^b^	−0.52^b^	−0.25	−0.03
Depression	.76	4.23	−4.00	2.00	−1.00	−2.00	4.00	−5.00	−5.00^b^	3.00	3.00	4.00
Anxiety	.84	5.15	−1.00	−3.00	2.00	−1.00	2.00	−3.00	−11.00^b^	2.00	5.00^b^	−6.00^b^
Psychiatric impairment	.84	3.10	−3.00	1.00	2.00	−4.00^b^	2.00	3.00	−9.00^b^	−2.00	1.00	1.00
Goal conflict reorganization	.89	13.23	12.70	9.10	−1.00	−6.60	14.20^b^	12.10	33.10^b^	11.40	9.90	−6.10
Self-efficacy	.81	3.27	5.00^b^	−3.00	0.00	0.00	1.00	−1.00	8.00^b^	−1.00	−4.00^b^	0.00
Problem-related distress	.66	3.98	−6.00^b^	−2.00	−1.00	−3.00	2.00	−8.00^b^	−10.00^b^	−3.00	−8.00^b^	2.00

^a^The values in the cells under each participant are the changes in their scores on each outcome measure from baseline to posttesting.

^b^Denotes reliable changes.

## Discussion

### Principal Findings

In this study, we developed a PWA version of MYLO through iterative co-design with a diverse group of young people from Western Australia. We established the feasibility of our research design to test MYLO by recruiting the target number of participants and reaching our diversity criteria with respect to gender, age, ethnicity, and geographical region. We successfully retained 77% (10/13) of the participants for the web-based surveys and qualitative feedback after 2 weeks. The retention rate, albeit for a short period, compares favorably with similar studies on mental health chatbots [21,24,62]. A good level of retention was consistent with the acceptability of the research design, with most measures rated as easy to complete. We established a good level of acceptability of the app in terms of use, ratings of system utility, therapeutic process, and helpfulness of MYLO’s questions, as well as gathering qualitative data and recommendations to improve MYLO in the future.

By analyzing MYLO’s use of search and question terms, we established that MYLO worked as it had been designed and could learn from users. We also obtained several interface design recommendations to implement in the next developmental stage. The effect sizes for the research measures over the 2-week period varied, but they showed sufficient promise to embark on a larger trial of effectiveness, with a longer intervention period and comparison condition.

By undertaking an iterative co-design phase, we were able to incorporate many user-led features and ensure that the interface was underpinned by expert insight. We have documented recommendations for further development of MYLO, which can also inform mental health chatbots more broadly. For example, users’ request for additional personalization and customization options aligns with previous user experience research, which found that young people prefer apps that they can be personalized and tailored to their needs [63,64]. Despite the growing number of digital mental health interventions and chatbots available for young people, few researchers are engaging with users to improve the effectiveness, uptake, and adherence rates of their innovations [65]. Of the 30 digital mental health technologies identified by Jones et al [65], only 2 papers reported on the co-design of a mental health chatbot [66,67]. By engaging in meaningful co-design and ensuring that MYLO’s interface is engaging and appealing to young people, we believe that we will be able to achieve high levels of retention and engagement with the app, which we would not have otherwise achieved, leading to improved clinical outcomes for users. We plan to test this hypothesis in a larger, fully powered trial.

Achieving a diverse sample is critical for assessing the acceptability of MYLO and the research design. Our findings suggest that MYLO is acceptable for a diverse range of young people. This builds on previous research, as studies involving real-world samples often provide very little information about participants [68]. Other studies have mostly included student populations [19,24,69], White people [24,70], and women [19,70].

Although most of the web-based measures were rated as easy to complete, the engagement measure and the SIS were rated as “neutral.” Given the apparent issues with participants retrospectively reporting their use of MYLO, these data will be collected directly from the app in the future. To ensure time efficiency, secure user identification was not implemented in this case series. However, this has now been established and will allow a range of anonymized user-specific metrics to be collected from MYLO and analyzed. With regard to the SIS, our findings suggest that MYLO is similar to other digitized mental health interventions [60] but currently performs slightly worse than face-to-face therapy [57,58,60]. Because very few studies have measured therapy or user satisfaction with mental health chatbots, there is no broadly accepted measure for this group of technologies. Using the SIS allowed us to examine the subscales of concepts relevant to MYLO (such as problem-solving) as well as identify where MYLO was performing well and where it could be improved. The session evaluation questionnaire [71] is also used to evaluate face-to-face therapy, but the subscales may be less applicable to chatbots or other digitized therapies that are user led. For example, the smoothness subscale may not fairly evaluate a chatbot or any therapy that does not follow an organized plan but is rather completely user led.

Another key improvement recommended by participants was the addition of a brief measure of adverse life events that occurred during the trial, as some participants experienced stressful life events during the trial that they felt may have impacted their clinical outcome scores. Many trials have already gathered information on adverse life events that occurred before the trial using a variety of measures [72,73]. A recent review [74] only identified 2 controlled trials of chatbots that gathered information on adverse events during the trial but specifically asked about harms caused by the chatbots [25,75]. To our knowledge, no studies have collected data on stressful life events that are not attributed to the intervention being investigated. The planned effectiveness trial will gather the usual safety information, that is, adverse events caused by MYLO, and allow participants to report other events in their life that may have impacted their clinical outcomes. This will allow us to see whether any confounding factors contributed to the results and also to see how MYLO is able to support people while they are experiencing different conditions and levels of stress.

The mixed methods approach to exploring the experience of using MYLO allowed us to gain a well-rounded and in-depth view. For example, we characterized the lengths and timing of conversations, discovered reasons for both its use and lack of use, and established MYLO’s successful use of search terms and related questions and its capacity to learn to adapt these weightings through user ratings. We identified a potential threshold of 1000 characters for a conversation with MYLO to be rated as helpful, as opposed to unhelpful or “neither.” Similarly, researchers found that a higher number of messages exchanged with another artificial intelligence chatbot was associated with more positive feedback [76], and increased engagement led to improvements in symptoms of anxiety and depression [77]. We will attempt to replicate this finding in the full trial and potentially use machine learning to identify the “signatures” of these “long and helpful” conversations. With secure anonymous user identification, tracking individual users across multiple conversations will further improve our understanding of the trajectory of “helpful” conversations.

Both qualitative analyses and quantitative data in this study provided insight into how MYLO was helpful. Participants’ ratings of the therapeutic process with MYLO were comparable with computerized cognitive behavioral therapy on several subscales, although generally less favorable than benchmarked brief, in-person psychotherapies. Most notably, MYLO seemed to approach in-person therapies in terms of ratings of how well it promotes understanding of a problem, but it scored lower in terms of the quality of the relationship. This is expected because MYLO is not currently programmed to try to foster a relationship with the user; rather, its primary aim is to promote the user’s understanding of a problem in greater depth and detail through curious questions. Consistent with this observation, participants reported that they would continue to ask themselves questions similar to those asked by MYLO after leaving a conversation. Indeed, some participants (3/8, 38%) found the lack of a human therapist to be advantageous. These findings are consistent with the theoretical principles of MYLO (PCT), which implies that everyone differs in what external therapeutic conditions allow the internal process of psychological change to occur [78].

Consistent with the abovementioned perspective, we found a small-to-moderate effect size for improvement in “reorganization of conflict,” the proposed mechanism of change, after the 2-week access to MYLO. The large effect size for reducing scores on the primary outcome (problem-related distress) supports this as the primary outcome measure for the planned effectiveness trial. This finding is consistent with earlier studies on the brief use of MYLO [41]. Similar to earlier brief interventions, we did not expect to find substantial effect sizes for clinical measures, and we did not. However, we recognize that these are only within-group effect sizes that have the usual potential biases (eg, maturation effects or attrition bias), but they do provide preliminary evidence for “promise” of MYLO to merit evaluation in an RCT. The planned effectiveness trial will also initially offer MYLO for 3 months rather than for 2 weeks and will use the version of MYLO that will incorporate many of the recommendations that have been generated from this case series and prioritized systematically. The acceptability and feasibility of collecting these clinical data remotely within this age group have, nonetheless, been established.

### Future Developments

Before the planned effectiveness trial, we will undertake further developments to address user concerns and recommendations. The largest issue raised by users was that sometimes MYLO’s questioning could become repetitive. This is an issue faced by many chatbots [79], and we believe it can be overcome by using natural language processing, such as Chat Generative Pretraining Transformer (ChatGPT). We plan to explore the use of a natural language processing platform that uses a bias engine specifically trained on mental health topics. Using this technology may improve the ability of MYLO to better understand and identify relevant terms in users’ conversations, thereby improving the helpfulness of questions throughout a conversation. Furthermore, this technology could allow MYLO to phrase questions in a variety of novel ways without requiring a very large database of questions.

To address the issue of some participants (4/8, 50%) not understanding the questions, we are exploring 2 strategies. First, we are planning to add a short introduction at the start of each conversation explaining the purpose of the questions and type of questions users can expect during a MYLO conversation. This is a technique used by some practitioners, A Churchman and N Gluckman (meeting, March 2023), when conducting the MOL therapy with young people to prime clients to be open to exploration. We are also exploring ways for users to prompt MYLO to rephrase a question when needed. This includes offering rephrasing and context to questions in tool tips or using a natural language processing platform to generate new questions with the same aim that might be simpler for users to understand.

To improve MYLO’s scores on the session impact subscales (understanding, problem-solving, relationship, and hindering impacts), we are planning to undertake another co-design and development phase to improve MYLO’s ability to support and understand young people. We anticipate that this will involve increasing the range of common problems faced by young people and the range of language (including slang) that MYLO is able to recognize and respond to [80]. We also hope to expand the range of ways MYLO responds, without changing MYLO’s goal of asking curious questions, to include encouragers [81] to help users feel understood [82].

This study has several limitations. First, the case series used a small sample. Therefore, we did not conduct any inferential statistics on the clinical outcome measures and could not make any substantial comments on MYLO’s effectiveness in improving the mental health of young people. The results of this study should be considered with caution, as it is possible that any effects found could be because of the natural recovery processes rather than an impact of MYLO. We aim to address this limitation in a larger trial. Second, the short 2-week follow-up time, although demonstrated a promising impact on problem-related distress, was unlikely to have an impact on anxiety and depressive symptoms. We will offer MYLO for a longer period and anticipate that prolonged decreases in problem-related distress will lead to improvements in anxiety and depression.

### Conclusions

In conclusion, we developed and tested the feasibility and acceptability of the newly developed version of MYLO, a mental health chatbot app, through iterative co-design with a diverse group of young people from Western Australia. By engaging in a meaningful co-design, the study was able to achieve high levels of retention and engagement, leading to improved clinical outcomes for users. Participants provided several interface design recommendations to further improve MYLO’s acceptability to be implemented in the next developmental stage, including additional personalization and customization options. Participants’ improvements in their ability to resolve internal conflicts and problem-related distress provided sufficient promise to embark on a larger trial of effectiveness with a longer intervention period.

## Appendix

Multimedia Appendix 1 Co-design iterative process.

Multimedia Appendix 2 Focus group topic guide.

Multimedia Appendix 3 Difficulty ratings.

Multimedia Appendix 4 Summary of themes and terms used in the testing phase.

Multimedia Appendix 5 Individual Session Impact Scale subscale scores.

## Abbreviations

MOL: method of levels
MYLO: Manage Your Life Online
PCT: perceptual control theory
PWA: progressive web application
RCT: randomized controlled trial
SF-6Dv2: Short Form-6D version 2
SIS: Session Impact Scale
