# Correction: An Artificial Therapist (Manage Your Life Online) to Support the Mental Health of Youth: Co-Design and Case Series

**DOI:** 10.2196/69628

**Published:** 2024-12-16

**Authors:** Aimee-Rose Wrightson-Hester, Georgia Anderson, Joel Dunstan, Peter M McEvoy, Christopher J Sutton, Bronwyn Myers, Sarah Egan, Sara Tai, Melanie Johnston-Hollitt, Wai Chen, Tom Gedeon, Warren Mansell

**Affiliations:** 1 Curtin enAble Institute Faculty of Health Sciences Curtin University Perth Australia; 2 Discipline of Psychology School of Population Health Curtin University Perth Australia; 3 School of Arts and Humanities Edith Cowan University Perth Australia; 4 Mental Health Commission of Western Australia Perth Australia; 5 Curtin Institute for Data Science Curtin University Perth Australia; 6 Centre for Clinical Interventions North Metropolitan Health Service Nedlands Australia; 7 Centre for Biostatistics, School of Health Sciences, The University of Manchester Manchester United Kingdom; 8 Alcohol, Tobacco and Other Drug Research Unit South African Medical Research Council Parow South Africa; 9 Division of Addiction Psychiatry Department of Psychiatry and Mental Health University of Cape Town Cape Town South Africa; 10 Department of Clinical Psychology, School of Health Sciences, The University of Manchester Manchester United Kingdom; 11 Mental Health Service, Fiona Stanley Hospital Perth Australia; 12 Curtin Medical School, Curtin University Perth Australia; 13 Centre of Excellence in Medical Biotechnology, Faculty of Medical Science, Naresuan University Phitsanulok Thailand; 14 Optus-Curtin Centre of Excellence in AI, School of Electronic Engineering, Computing and Mathematical Sciences, Curtin University Perth Australia

In “An Artificial Therapist (Manage Your Life Online) to Support the Mental Health of Youth: Co-Design and Case Series” (JMIR Hum Factors 2023;10:e46849) the authors noted an error.

In the Results section of the text, a variable was wrongly described as the number of words typed by users, instead of the number of characters typed by users. Therefore, all mention of this variable has been revised.

The following sentence:

The word count of these conversations ranged from 58 to 2104 words and participants sent 2 to 20 texts.

has been revised to:

The character count of these conversations ranged from 58 to 2104 characters including spaces and participants sent 2 to 20 texts.

The following sentence:

As shown in Figure 3, in total 100% of the conversations 1000 words were rated as helpful, and all the remaining conversations 1000 words were rated as either unhelpful or neither.

has been revised to:

As shown in Figure 3, in total 100% of the conversations in which participants typed over 1000 characters were rated as helpful, and the remaining conversations were rated as either unhelpful or neither.

The caption and axis title of Figure 3 have also been revised from:

Figure 3. Number of participant-generated words in each conversation and overall helpfulness rating.

to:

Figure 3. Number of participant-generated characters in each conversation and overall helpfulness rating.

In the discussion section the following sentence:

We identified a potential threshold of 1000 words for a conversation with MYLO to be rated as helpful, as opposed to unhelpful or “neither.”

has been revised to:

We identified a potential threshold of 1000 characters for a conversation with MYLO to be rated as helpful, as opposed to unhelpful or “neither.”

The correction will appear in the online version of the paper on the JMIR Publications website on December 16, 2024, together with the publication of this correction notice. Because this was made after submission to PubMed, PubMed Central, and other full-text repositories, the corrected article has also been resubmitted to those repositories.

