# Supplementary material for: An Artificial Therapist (Manage Your Life Online) to Support the Mental Health of Youth: Co-Design and Case Series
Source: JMIR Hum Factors. 2023 Jul 21;10:e46849. doi: 10.2196/46849 (PMC10403793; doi:10.2196/46849)
Supplement: Multimedia Appendix 1 [file humanfactors_v10i1e46849_app1.pdf]

Multimedia Appendix – Codesign Iterative Process

This is a Multimedia Appendix to a full manuscript under review in the JMIR.

Youth Advisory Meetings, Feedback Received and How it was Implemented with Screenshots of Each Iteration of MYLO

Date	1st July 2022
Consumer Feedback	Consumers raised concerns about gaining parental consent.
How it was implemented	The option of not requiring parental consent was explored and ultimately the study protocol was changed to allow under 18's to opt out of providing parental consent, if they demonstrated they understood the study and what participation entailed as well as providing their reasoning for not involving their parents.

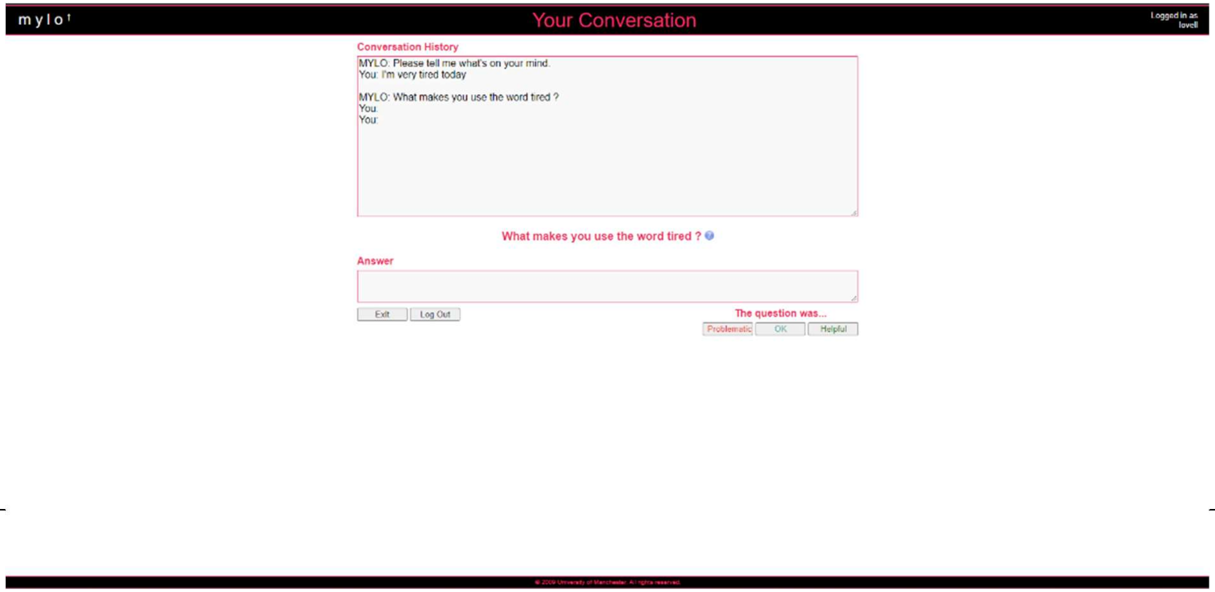

Date	29th July 2022
Consumer Feedback	Consumers liked the simplicity of the interface and talking to MYLO (they found it similar to journalling). Features suggested: access to more resources in the app, an introduction or instructions on what MYLO does and when to use it, personalisation options, option to save a profile.
How it was implemented	Based on other messaging apps and user feedback from previous case studies of MYLO and the current user feedback a chat box was created. To make the user’s messages and MYLO’s distinct messages more obvious they we’re done as separate colours. Also, as per user feedback a profile was created which gets stored on the device. This profile also stores the user themes, the images provided are in dark mode Additional Mental health Resources and Lifeline was added as a button. The Lifeline button when pressed calls Lifeline.

<table>
<tr><td colspan="2">
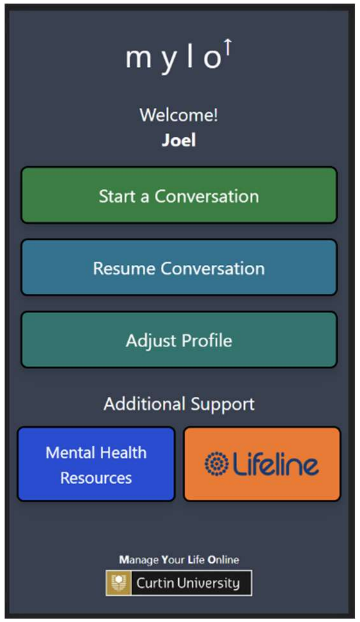

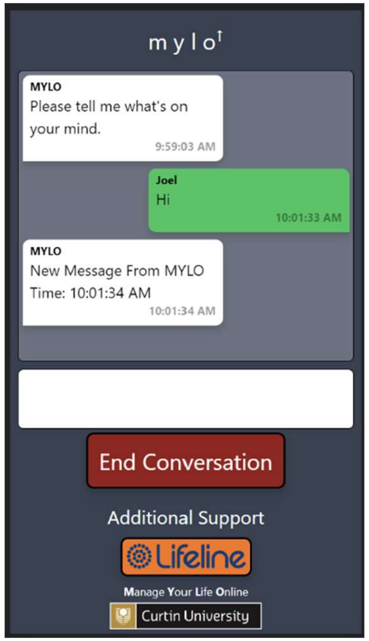

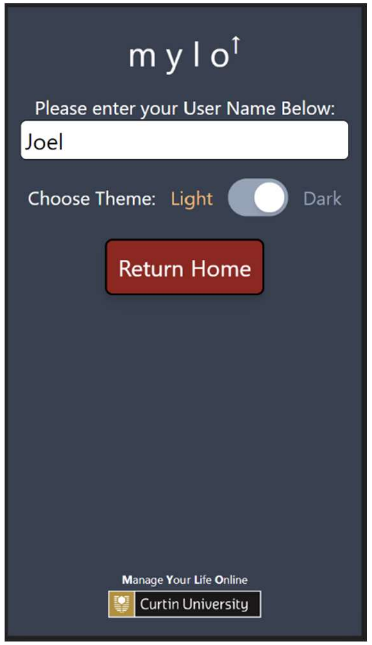

</td></tr>
<tr><td>Date</td><td>25th August 2022</td></tr>
<tr><td>Consumer Feedback</td><td>Consumers provided feedback on the new interface, the MYLO logo and suggestions to improve MYLO. Consumers commented on some of the colours used in the interface and wording of buttons. Consumers were concerned that the interface was accessible for vision-impaired and colour-blind people.</td></tr>
<tr><td>How it was implemented</td><td>As per previous feedback, the profile was expanded to offer more customization options for users to help personalize the app. As per user feedback an avatar chosen by the user was created for the chat, this avatar is</td></tr>
</table>

	customizable. The username now appears on all messages. There is an option to resume the most recent conversation as well as per user request. This was a feature of the previous version of MYLO and thus needed to be implemented either way for feature parity with the original MYLO. The backend was being developed at this time waiting for user feedback on the UI. The changes
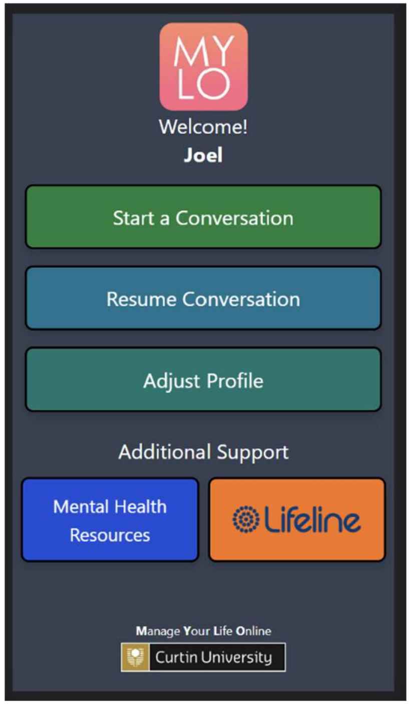 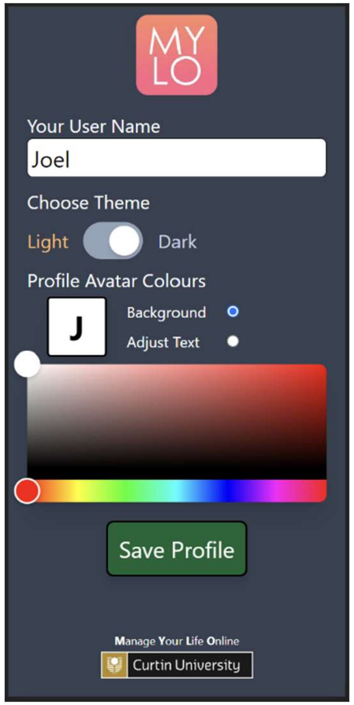 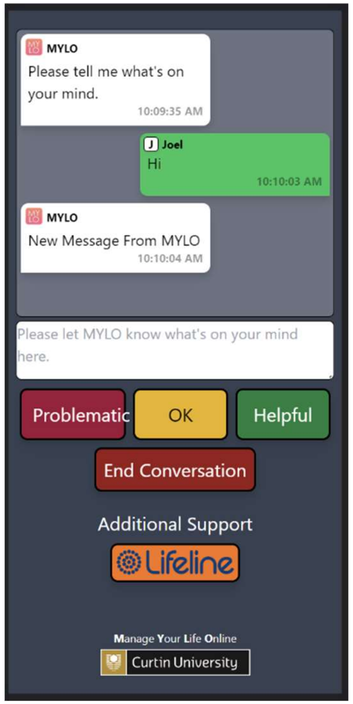	
Date	14th October 2022

Consumer Feedback	Consumers were shown the new logo, the new interface and the new website instructions in preparation for the case series starting 17th October.
How it was implemented	To address the colour blindness issues from the previous feedback, we consulted with the graphics design team at the CIC and used the new logo for the basis of a new colour theme. This new logo incorporated the arrows from the previous logo to which many users preferred. Accessibility tools built into Chrome Dev Tools and other development tools such as WAVE and aXe were used to check the colour contrasts were 4.5:1 as per the accessibility guidelines. Many UI elements were created to introduce images to help users go through the app intuitively, instructions were created to help new users use the app.<br><br>To address some of the user feedback concerning in conversation prompts and the ending of the conversation, there is a way to rate the conversation as Helpful, Neither or Unhelpful, once user clicks End Conversation. The logo is now consistent amongst both dark and light themes with the only change occurring to the “mylo” font from black to white for readability.<br><br>To further address the needs for further resources, the hamburger menu was created to contain the instructions and mental health resources links inside.

<table>
<tr><td></td><td>General performance improvements and the backend API which returns the new question amongst other things was also ready to go and very responsive.</td></tr>
<tr><td colspan="2">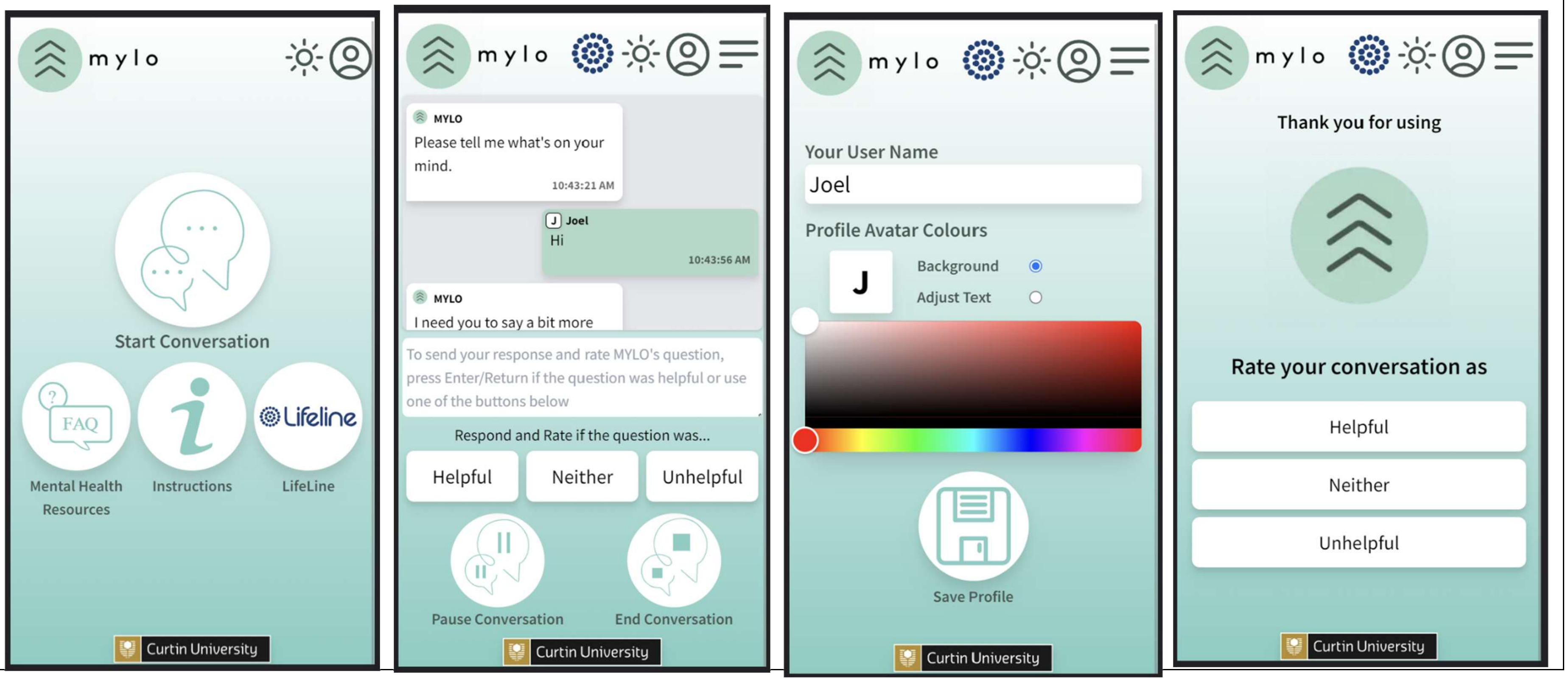
</td></tr>
</table>

Date	19th October 2022
Consumer Feedback	The Case Series had started and there were some users who had issues with the accessibility of the app as those with vision impairment would set the size of the font to be much larger than the default font sizing while also using smaller screens than what MYLO had been tested on. Users also requested a bit more feedback is given when buttons are touched or clicked on. Users also requested more mental health resources be accessible from the app.
How it was implemented	To address the accessibility issues the number of on-screen elements in the navbar changed to two during a conversation moving much of the navbar resources into the hamburger menu. The sizing of elements and fonts across the app was changed slightly to improve the accessibility for those who use larger fonts ensuring elements are still visible on the screen.<br>All buttons now have subtle animations changing in size or colour when pressed or hovered over to give users the feedback they desired when pressing any button. To increase accessibility, users who might prefer less animation by activating the preferred-less-motion setting in the browser are also catered for as all the animations are turned off in the case.

	More Mental Health Resources such as Headspace and Beyond Blue were added as direct links in the app. The Curtin university logo now goes to the mylochat.com site as well acting like the About MYLO link in the hamburger menu.<br>There was also an issue with the Profile page, the save profile button would appear even if the username was blank. That was fixed while also making it more apparent to those with colour blindness that the username was missing.

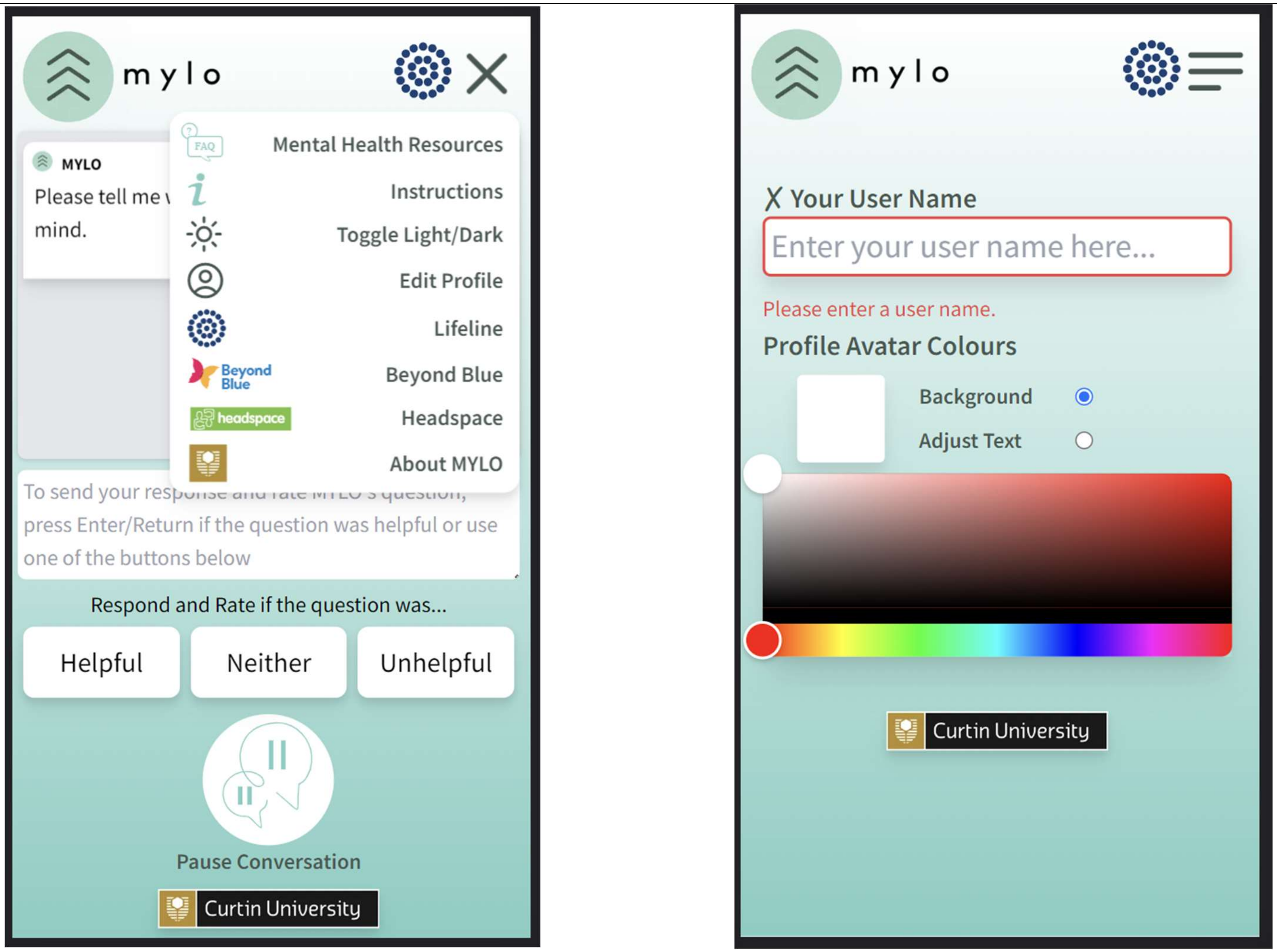
mylo
MYLO
Please tell me
mind.
Mental Health Resources
Instructions
Toggle Light/Dark
Edit Profile
Lifeline
Beyond Blue
Headspace
About MYLO
To send your response and rate MYLO's question, press Enter/Return if the question was helpful or use one of the buttons below
Respond and Rate if the question was...
Helpful
Neither
Unhelpful
Pause Conversation
Curtin University
mylo
X Your User Name
Enter your user name here...
Please enter a user name.
Profile Avatar Colours
Background
Adjust Text
Curtin University
