# Supplementary material for: An Artificial Therapist (Manage Your Life Online) to Support the Mental Health of Youth: Co-Design and Case Series
Source: JMIR Hum Factors. 2023 Jul 21;10:e46849. doi: 10.2196/46849 (PMC10403793; doi:10.2196/46849)
Supplement: Multimedia Appendix 2 [file humanfactors_v10i1e46849_app2.docx]

**Multimedia Appendix – Focus Group Topic Guide**

**This is a Multimedia Appendix to a full manuscript under review in the JMIR.**

Most questions adapted from Gaffney et al., 2018, questions noted with an* adapted from Ly et al., 2012.

Welcome - Acknowledgements of country and lived experience.

Introductions: Research Team

Purpose of the focus group: You have been asked to participate in this focus group to provide your feedback on your experience of testing MYLO for the last two weeks. The information learned in this focus group will be used to improve the MYLO interface and the study protocol for a larger trial planned for next year.

Ground rules:

- You can choose whether or not to participate in the focus group, and if you need to you can stop at any time by leaving the meeting.

- The focus group will be recorded, but your responses will be deidentified once a written copy of the recording is made, and then the recording will be deleted. You do not need to have your camera on if you are not comfortable doing so.

- There are no right or wrong answers to the question we are going to ask you. We are interested in everyone’s honest opinions, even if they are different to others in the group or are negative of MYLO.

- We ask that everyone respects each other, and as best as possible we would like one person to speak at a time.

- We would like to ask that everyone does not repeat what is discussed today, to maintain confidentiality of the group.

- However, it is always a possibility with focus groups that participants repeat what is discussed, so we would ask please do not disclose any sensitive information.

- If it appears you might be about to disclose sensitive information, one of the research team may interrupt you to remind you of the limits of confidentiality.

- If you do want to share feedback that includes sensitive information there will be time after the focus group to do so, once other participants have left the meeting.

- Be advised that in some instances where reportable incidents are shared (such as child sexual abuse or other risk issues) the researchers will share what has been disclosed to relevant bodies. You will be consulted if this needs to occur after the focus group.

Questions

1) How easy was it for you to use MYLO?

a) How often did you use MYLO?*

b) When (in what situations) did you use MYLO? Or would you if you had longer access to the app?*

c) What was your general experience of using MYLO?*

d) What made it easier/harder to use?

e) What suggestions do you have to make MYLO easier to use?

2) What did you think of the look of MYLO?

a) Could you tell us about anything you would change?

b) Are there things you thought worked well about the design?

3) What was your experience of typing out your problems to MYLO?

a) In what ways were you able to explore your problems using MYLO?

b) Can you tell me about the things you might have found difficult about the text conversation?

c) Is there anything you particularly liked about text conversations with MYLO?

4) Do you have any suggestions on how MYLO may be improved?

5) What are your thoughts on whether you would recommend MYLO to others?

a)Why would you / would not

6) What was your experience of completing the assessments?

a) How acceptable did you find them to complete?

b) Were any of the questions in the assessments hard to answer?

c) What suggestions do you have for improving the assessments?

Thank all the participants for their time and expertise. Explain they will receive their gift vouchers by email within 24 hours.
