# Supplementary material for: An Artificial Therapist (Manage Your Life Online) to Support the Mental Health of Youth: Co-Design and Case Series
Source: JMIR Hum Factors. 2023 Jul 21;10:e46849. doi: 10.2196/46849 (PMC10403793; doi:10.2196/46849)
Supplement: Multimedia Appendix 3 [file humanfactors_v10i1e46849_app3.pdf]

Multimedia Appendix – Difficulty Ratings

This is a Multimedia Appendix to a full manuscript under review in the JMIR.

Number of Participants who Provided Each Difficulty Rating for each Questionnaire at Post-Testing and Mean Rating Scores

Questionnaire	Very difficult	Difficult	Neutral	Easy	Very Easy	*M (SD)*
General Health	0	1	4	2	3	0.7(1.06)
Depression	0	1	4	2	3	0.7(1.06)
Anxiety	0	2	3	2	3	0.6(1.17)
Psychiatric impairment	0	2	2	3	3	0.7(1.16)
Goal conflict reorganisation	0	1	3	3	3	0.8(1.03)
Self-efficacy	0	2	3	2	3	0.6(1.17)
Problem related distress	0	2	3	2	3	0.6(1.17)
Therapeutic satisfaction	0	3	2	4	1	0.3(1.06)
System usability	0	2	2	4	2	0.6(1.08)
Engagement	1	1	2	5	1	0.4(1.17)

Note. Scale from -2 to 2, with higher scores indicating higher ease.
