# Supplementary material for: An Artificial Therapist (Manage Your Life Online) to Support the Mental Health of Youth: Co-Design and Case Series
Source: JMIR Hum Factors. 2023 Jul 21;10:e46849. doi: 10.2196/46849 (PMC10403793; doi:10.2196/46849)
Supplement: Multimedia Appendix 4 [file humanfactors_v10i1e46849_app4.docx]

**Multimedia Appendix – Summary of Themes and Terms Used in the Testing Phase**

**This is a Multimedia Appendix to a full manuscript under review in the JMIR.**

| Summary of the Themes and Terms Used in the Testing Phase | | |
| --- | --- | --- |
| Theme | Example of terms used by participants | Frequency |
| Feelings | “Worried”, “happy”, “anxious”, “sad” | 19 |
| Inability | “I do not know how to”, “like I cannot” | 6 |
| Conflict | “Pressure”, “overwhelmed”, “effort” | 5 |
| Events future | “The future”, “it will” | 4 |
| Want to | “I want to”, “I wish” | 6 |
| Anger | “Infuriated”, “annoyed” | 2 |
| Apprehension | “Uncertain”, “not sure” | 4 |
| Sense of | “I feel like”, “feeling of” | 7 |
| Balance | “Unsure” | 1 |
| Cognitive dissonance | “Maybe” | 1 |
| Control | “I cannot control” | 1 |
| Emotive term | “Gender” | 1 |
| Goal progress | “A little better” | 2 |
| Helped | “Helps me” | 1 |
| Images | “I see” | 1 |
| Medical | “Heart rate increases” | 1 |
| No motivation | “Exhausted” | 1 |
| Personal judgements | “I am worthless” | 1 |
| Person label | “Bored” | 1 |
| Rumination | “Thinking about things” | 1 |
| Someone to talk to | “Someone to talk to” | 1 |
| Time | “Not enough time” | 1 |
| Work | “Duties” | 1 |
