# Supplementary material for: An Artificial Therapist (Manage Your Life Online) to Support the Mental Health of Youth: Co-Design and Case Series
Source: JMIR Hum Factors. 2023 Jul 21;10:e46849. doi: 10.2196/46849 (PMC10403793; doi:10.2196/46849)
Supplement: Multimedia Appendix 5 [file humanfactors_v10i1e46849_app5.docx]

**Multimedia Appendix – Individual SIS Subscale Scores**

**This is a Multimedia Appendix to a full manuscript under review in the JMIR.**

| **Participants Mean SIS Subscale Scores for MYLO** | | | | | | | | | | | |
| --- | --- | --- | --- | --- | --- | --- | --- | --- | --- | --- | --- |
|  | **Understanding** | | **Problem-Solving** | | **Relationship** | | **Hindering** | | **Unwanted Thoughts** | |  |
| **Participant** | ***M*** | ***SD*** | ***M*** | ***SD*** | ***M*** | ***SD*** | ***M*** | ***SD*** | ***M*** | ***SD*** |  |
| **1** | **2.33** | **0.47** | **1.75** | **0.35** | **2.00** | **0.00** | **1.50** | **0.24** | **1.50** | **0.71** |  |
| **2** | **2.67** | **0.47** | **1.75** | **0.35** | **2.50** | **0.71** | **2.58** | **0.12** | **2.00** | **0.00** |  |
| **3** | **3.50** | **0.71** | **4.00** | **0.00** | **3.40** | **0.57** | **1.75** | **0.12** | **1.00** | **0.00** |  |
| **4** | **1.83** | **0.24** | **1.25** | **0.35** | **2.20** | **0.28** | **2.08** | **0.12** | **2.00** | **0.00** |  |
| **5** | **2.33** | **0.47** | **3.00** | **0.00** | **2.90** | **0.71** | **1.17** | **0.00** | **1.50** | **0.71** |  |
| **6** | **2.00** | **0.47** | **1.75** | **0.35** | **1.50** | **0.42** | **1.33** | **0.24** | **1.00** | **0.00** |  |
| **7** | **4.00** | **0.00** | **3.75** | **0.35** | **3.90** | **0.42** | **1.08** | **0.12** | **1.50** | **0.71** |  |
| **9** | **1.83** | **0.71** | **1.75** | **1.06** | **2.20** | **0.00** | **2.42** | **0.59** | **2.50** | **2.12** |  |
| **10** | **1.00** | **0.00** | **1.00** | **0.00** | **1.00** | **0.00** | **2.33** | **0.71** | **1.00** | **0.00** |  |
| **11** | **3.50** | **0.71** | **2.00** | **0.00** | **1.80** | **0.28** | **1.58** | **0.59** | **1.50** | **0.71** |  |
| **12** | **1.00** | **-** | **1.00** | **-** | **1.00** | **-** | **1.50** | **-** | **1.00** | **-** |  |
| **Note. Only participants who completed at least one survey containing the SIS are included.** | | | | | | | | | | | |
